# Towards Low Temperature Operation of Catalytic Gas Sensors: Mesoporous Co_3_O_4_-Supported Au–Pd Nanoparticles as Functional Material

**DOI:** 10.3390/nano13152192

**Published:** 2023-07-27

**Authors:** Xuemeng Lyu, Haitao Gao, Patrick Diehle, Frank Altmann, Katrin Schmitt, Karina Tarantik, Jürgen Wöllenstein

**Affiliations:** 1 Department of Microsystems Engineering (IMTEK), University of Freiburg, 79110 Freiburg, Germany; xuemeng.lyu@ipm.fraunhofer.de (X.L.);; 2 Fraunhofer Institute for Physical Measurement Techniques (IPM), 79110 Freiburg, Germany; 3 Fraunhofer Institute for Microstructure of Materials and Systems (IMWS), 06120 Halle, Germany

**Keywords:** catalytic gas sensor, pellistor, methane, low-temperature operation, mesoporous Co_3_O_4_, Au–Pd nanoparticles

## Abstract

It is shown that the operating temperature of pellistors for the detection of methane can be reduced to 300 °C by using Au–Pd nanoparticles on mesoporous cobalt oxide (Au–Pd@meso-Co_3_O_4_). The aim is to reduce possible catalyst poisoning that occurs during the high-temperature operation of conventional Pd-based pellistors, which are usually operated at 450 °C or higher. The individual role of Au–Pd as well as Co_3_O_4_ in terms of their catalytic activity has been investigated. Above 300 °C, Au–Pd bimetallic particles are mainly responsible for the catalytic combustion of methane. However, below 300 °C, only the Co_3_O_4_ has a catalytic effect. In contrast to methane, the sensor response and the temperature increase of the sensor under propane exposure is much larger than for methane due to the larger heat of combustion of propane. Due to its lower activation energy requirement, propane exhibits a higher propensity for oxidation compared to methane. As a result, the detection of propane can be achieved at even lower temperatures due to its enhanced reactivity.

## 1. Introduction

The early detection of flammable and explosive gas mixtures is highly significant for avoiding danger to people as well as damage to infrastructure. Millions of catalytic sensors (so-called pellistors) for this application are being installed annually [1,2,3,4,5]. Conventional pellistors work at high temperatures (>450 °C), ensuring that a catalytic reaction occurs even in the case of the gases which are difficult to oxidize such as methane [6,7,8,9,10,11]. The gas sensitive layer of commercially available sensors very often consists of palladium-coated porous Al_2_O_3_. Taking methane for example, it is relevant, from a safety point of view, to detect the gas reliably below 10% of the lower explosion limit (LEL) which is around 4% (i.e., 0.44% methane in air [12,13]). However, the measuring principle has some intrinsic disadvantages, such as poor stability, a small measuring range, and high susceptibility to catalyst poisoning [14,15]. The investigation of catalytic poisoning caused by silicon-containing gases, such as siloxanes, is essential due to their presence as trace contaminants in biogas, which can lead to irreversible inhibition of catalytic oxidation and sensor failures [1,16]. As an example, hexamethyldisiloxane (HMDSO) is stable up to a temperature of 300 °C, yet at temperatures above 300 °C, silicon dioxide is increasingly formed on the surface of the pellistor by the decomposing HMDSO, which passivates the functional material [17].

In order to minimize possible catalyst poisoning, it is, therefore, necessary to lower the operating temperature of the pellistors down to or even below 300 °C. This necessitates the exploration of innovative material systems that not only exhibit large surface areas or high porosity, but also display robust resistance against catalytic poisoning. An increasing number of studies are currently focused on the objective of reducing the operating temperature of pellistors [18]. The majority of palladium-based catalysts, while active for methane oxidation, typically encounter issues of poor stability and low tolerance to poisons. These challenges can be addressed by introducing a second metal to create a bimetallic or alloyed catalyst, or by implementing alternative synthesis methods such as electron-beam evaporation [19]. In contrast, bimetallic nanoparticles, which combine the electrochemical properties of their components have often demonstrated superior catalytic or sensing performance compared to monometallic nanoparticles due to synergetic effects [20,21,22]. Their improved performance is related to electronic and structural properties [23,24,25]. In addition, recent publications proved a higher stability against poisoning of bimetallic nanoparticles compared to monometallic nanoparticles [26,27,28,29]. For instance, the alloying Pd with Au or Pt showed distinct enhancement of the catalysts’ performance [30,31,32,33]. Alloys of Pd and Au also showed improved catalytic performance for methane combustion [31,32]. For example, Au–Pd alloy nanoparticles with an average size of 3.3 nm uniformly dispersed on the surface of the CoCr_2_O_4_ showed combustion of methane turnover rates of T10%, T50%, and T90% at 305 °C, 353 °C, and 394 °C, respectively, where T10%, T50%, and T90% represent the temperatures at which the catalytic reaction achieves 10%, 50%, and 90% of its conversion. The excellent catalytic performance was associated with higher surface area and adsorbed oxygen species concentration, better low-temperature reducibility, and strong interaction between Au–Pd alloy nanoparticles and CoCr_2_O_4_. An explanation for the better activity of the supported Au–Pd bimetal catalysts might be that Au could isolate the Pd sites within the bimetallic system. Hutchings et al. found that Au could act as an electronic promoter for Pd and the catalyst active for alcohol oxidation possessed a surface significantly enriched by Pd. The surface-bound oxygen centered radicals played a positive role in activating toluene over the supported Au–Pd alloy catalysts [34]. This was verified by Xie et al. [35]. In their study, the Pd^2+^/Pd^0^ molar ratios of the Mn_2_O_3_-supported Au–Pd alloy samples (1.15–1.7) were higher than that of Pd/Mn_2_O_3_ (0.97), suggesting that the addition of Au could enhance the concentration of Pd^2+^ species. The improvement of catalytic performance is not solely based on bimetallic composition of catalytic nanoparticles. The oxidic carrier material also has a major influence on the behavior, namely the morphology (surface, porosity) and the type of metal oxide used (e.g. strong interactions between alloy particles and support, coexistent species with different valence). Recently, Wu et. al. reported that mesoporous Co_3_O_4_ was used as a support for a bimetallic Au–Pd catalyst (Au–Pd@Co_3_O_4_) to improve the catalytic properties for methane combustion [32]. The excellent catalytic performance can be associated with coexistence of different valences in Co_3_O_4_. Among the transition-metal oxide catalysts, cobalt oxides show the highest activity in catalytic combustion. Even for resistive metal–oxide gas sensors, it has already been shown that the operating temperatures required for the detection of reactive gases of cobalt oxide-based sensors are significantly lower in comparison to other semiconducting metal oxides [36,37]. Since the Co^2+^ and Co^3+^ species coexist in Co_3_O_4_, the redox process can take place easily between Co^2+^ and Co^3+^ during methane combustion [38,39]. Wu’s work also described some material systems that achieved 50% methane conversion at 280 °C and 90% at 324 °C. In fact, these results are very close to the temperature at which silicon-containing gases remain stable (below 300 °C). Mesoporous Co_3_O_4_ impregnated with Au–Pd is, therefore, a promising functional material to build pellistors with significantly lower operating temperatures. In this contribution, we present our research on Au–Pd-supported meso-Co_3_O_4_ as a prospective solution to reduce the permanent catalytic poisoning that occurs during high-temperature operation of conventional Pd-based pellistors. These pellistors are typically operated at temperatures of 450 °C or higher. Our aim is to investigate the feasibility of using Au–Pd nanoparticles on mesoporous cobalt oxide to lower the operating temperature of pellistors for methane detection to 300 °C.

In this study, we utilized Au–Pd supported meso-Co_3_O_4_ as the key material for our investigation on reducing catalytic poisoning in pellistors and lowering the operating temperature for methane detection. The meso-Co_3_O_4_ catalyst served as the support material, while Au–Pd nanoparticles were incorporated to enhance the catalytic activity. The gas measuring station consisted of a specialized setup that allowed us to accurately measure the sensor response and temperature increase under different gas exposures. This station provided precise control of gas concentrations and temperature conditions, enabling reliable characterization of the performance of the Au–Pd@meso-Co_3_O_4_ pellistors. By employing these materials and utilizing the gas measuring station, we conducted a thorough analysis to understand the catalytic behavior and temperature dependence of the pellistors.

## 2. Materials and Methods

### 2.1. Synthesis of Functional Layers

**Figure 1:**
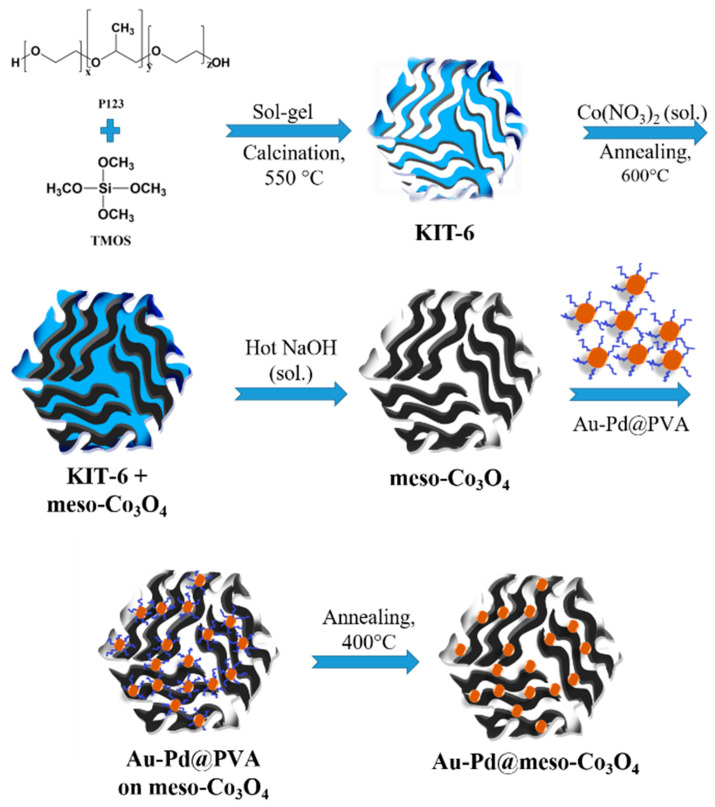
Synthesis of the functional material. Mesoporous KIT-6 silicon dioxide serves as a template, which was subsequently filled with Co_3_O_4_. After removing the silica, mesoporous cobalt oxide is formed on which Au–Pd-alloy nanoparticles are deposited.

The synthesis process of mesoporous Co_3_O_4_ impregnated with Au–Pd nanoparticles is illustrated schematically in Figure 1. The motivation for the synthesis described below was to make practical use of the promising results of the previously mentioned publication on Au–Pd impregnated mesoporous Co_3_O_4_ [32].

Three-dimensional mesoporous KIT-6 silica was prepared in an autoclave as a template [24]. Subsequently, Co(NO_3_)_2_·6H_2_O was mixed with KIT-6 in toluene. After filtration, the solid mixture was calcined at 600 °C for 4 h, leading to a formation of Co_3_O_4_ in the pores. The silica template was removed with hot NaOH at 70 °C and mesoporous Co_3_O_4_ was obtained. This was added to an Au–Pd alloy colloid solution (Au–Pd@PVA) prepared by reduction of Au and Pd salts (Au:Pd = 1:2) using NaBH_4_ in dilute polyvinyl alcohol solution (PVA). Extensive studies have been conducted on the Au–Pd ratio, and it has been demonstrated that a ratio of 1:2 exhibits the optimal catalytic ability [26,32,40]. The PVA serves as a surface stabilizer. After filtration, the Au–Pd@PVA@meso-Co_3_O_4_ material was annealed at 400 °C, where Au–Pd@meso-Co_3_O_4_ formed after thermal removal of the PVA. In the experiments described below, it is always material with a nominal wt12% Au–Pd content. In this study, 12% is was chosen since it is empirically the optical nominal value that can be loaded on mesoporous Co_3_O_4_ [32,41]. 

**Figure 2:**
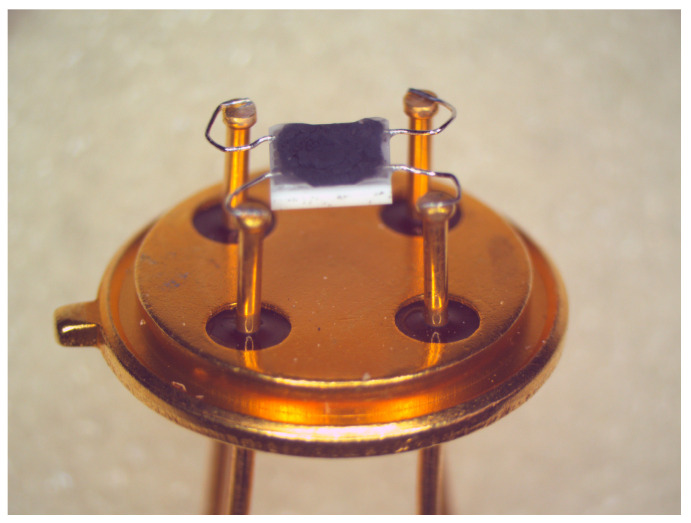
Ceramic substrates with deposited Au–Pd-impregnated mesoporous Co_3_O_4_. The sensor chips were suspended by Pt wires by means of micro-welding on a TO39 socket (ø 9.2 mm) to ensure a good thermal insulation of the sensor chip.

The final products were dispersed in deionized water with a solid content of 15 g/L to obtain a printable ink. The prepared ink was deposited on a Al_2_O_3_ substrate (2 mm × 2 mm) using a micropipette (0.2 μL/layer). Depending which layer thickness is desired, the deposition was repeated. The ceramic substrates are coated on the back with a platinum thin-film element, which simultaneously serves as a heating resistor and as a temperature sensor (Figure 2). The platinum thin-film element is patterned on the Al_2_O_3_ substrate using a standard MEMS technique. Finally, a thin layer of glass is printed on the backside platinum layer as a passivation. Platinum was chosen because of its linear temperature coefficient of 3850 ppm 1/K [42]. Al_2_O_3_ is applied not only due to its high thermal conductivity of 25 W/(mK), but also its thermal and chemical stability [43]. The catalytically functional layer was deposited on the front side. On the front side of the substrates are finger-shaped platinum electrodes for resistance measurements, which are not passivated and could have a small catalytic effect. However, the influence of these platinum electrodes is neglected in the following considerations because they are largely covered by the mesoporous Co_3_O_4_.

### 2.2. Material Characterization

**Figure 3:**
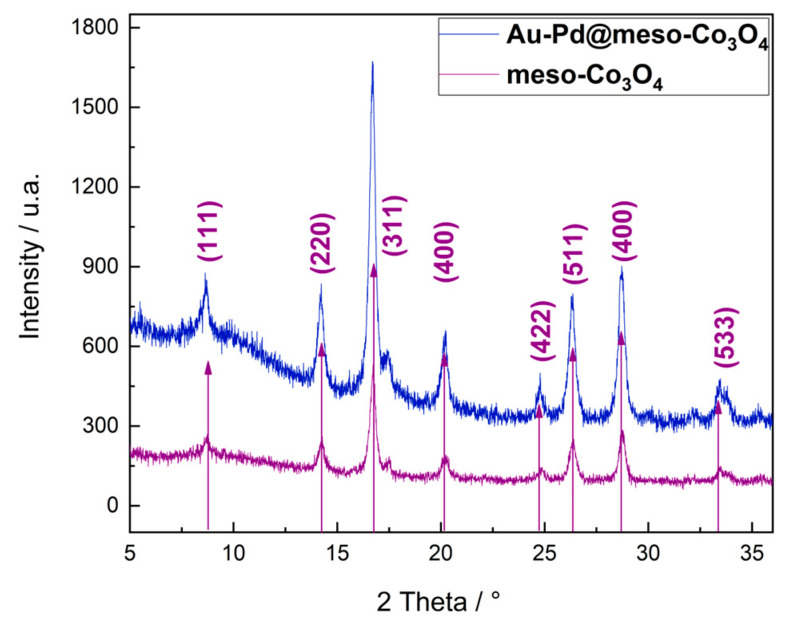
XRD results for Au–Pd@meso-Co_3_O_4_ (blue) and meso-Co_3_O_4_ (violet). In both cases, the measurement results show the typical cubic structure for crystalline cobalt (II, III) oxide. The indices correspond to Co_3_O_4_ and are based on reference JCPDS 42-1467. The Au–Pd particles cannot be seen in the measurement results, although the TEM investigations indicate that they are crystalline. The lack of a clear additional signal corresponding to the Au–Pd nanoparticles is probably due to the small size of the particles.

Powder XRD measurements were performed for mesoporous Co_3_O_4_ and Au–Pd (12 wt%)@meso-Co_3_O_4_ (nominal Au–Pd content). The powder XRD data (Figure 3) showed that the mesoporous Co_3_O_4_ (reference JCPDS 42-1467) is phase pure and has an average grain size of 12.1 nm, which was calculated using the Scherrer equation. The measurement of Au–Pd (12 wt%)@meso-Co_3_O_4_ shows also the Co_3_O_4_ peaks but no signal that clearly corresponds to the Au–Pd nanoparticles. Most likely the Au–Pd nanoparticles are not detectable due to reflection broadening caused by the small grain size of the particles. From SEM–EDX analysis on the samples made with 12 wt% Au–Pd, only a concentration of 7.5 wt% was determined, indicating a substantial loss during filtering.

**Figure 4:**
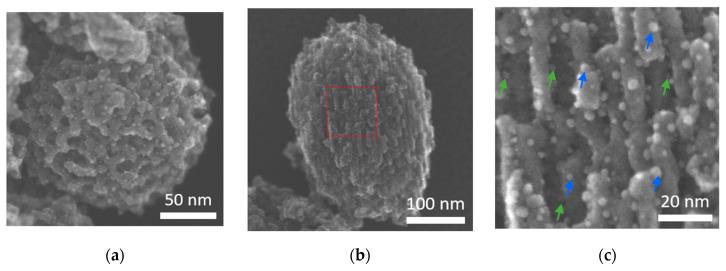
SE–STEM images of Au–Pd particles (nominal, 12 wt%) on mesoporous Co_3_O_4_. (**a**,**b**) show two different mesoporous structures. (**c**) shows a magnified section of (**b**), which is marked by a red square. The Au–Pd-particles are located on the surface (blue arrows) and inside the pores (green arrows).

The porous structure of Co_3_O_4_ on the one hand and the three-dimensional distribution of the Au–Pd particles on the other hand are of special interest regarding the catalytic properties of the material. Therefore SE–STEM measurements (Hitachi HF 5000, Hitachi, Tokyo, Japan) were performed at 200 kV (Figure 4) on Au–Pd (12 wt%)@meso-Co_3_O_4_, since the SE (secondary electron) signal contains surface information. Figure 4a,b show larger mesoporous structures, which reveal a symmetric structure and lead to the assumption that they consist of a three-dimensional network. Ordered and open pores have been observed in both structures, and the Au–Pd particles are uniformly distributed over the structure. Figure 4c reveals that the Au–Pd particles are located inside the pores as well as on the surface (indicated by green and blue arrows). The average particle size of Au–Pd particles was determined to be 3.13 ± 0.62 nm.

**Figure 5:**
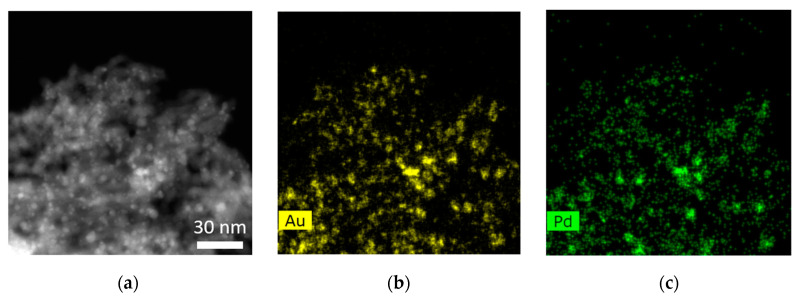
EDX analysis of Au–Pd particles (12 wt%) on mesoporous Co_3_O_4_. (**a**) shows the HAADF–STEM image of the sample. (**b**,**c**) show the elemental distribution of Au and Pd.

Additionally, STEM–EDX experiments (FEI Titan3 G2 60-300 equipped with FEI Super-X detector, FEI Company, Eindhoven, The Netherlands) were performed in order to analyze the chemical composition of the Au–Pd-particles (Figure 5). Figure 5b,c show that the elemental distributions of Au and Pd coincide. This indicates that no phase separation is taking place on the Au–Pd alloy particles.

### 2.3. Methods of Gas Sensitive Characterization

To perform gas-dependent characterizations, the pellistors were prepared by applying 15 layers of dispersed Au–Pd on meso-Co_3_O_4_ onto the substrates using a micropipette with a volume of 0.2 µL per layer. The selection of the optimal number of layers is crucial to achieve uniformity, efficient heat conduction, and proper combustion reaction in the catalytic process. An excessive number of layers can result in poor uniformity, while too few layers can lead to insufficient sensitive material, compromising the signal quality. After extensive optimization, we have determined that 15 layers strike the ideal balance, ensuring optimal performance and high-quality signals. After annealing at 400 °C for 12 h, a spherical layer was formed with a thickness about 20 µm at the rim and 120 µm in the middle. 

The experimental protocol involved positioning two pellistors within the configuration of a Wheatstone bridge, thereby ensuring precise measurement outcomes. The reference sensor in the bridge was a blank Al_2_O_3_ substrate with Pt heater. The other Al_2_O_3_ substrate coated with the catalytic functional material served as the active, gas-sensitive sensor. The sensor response is the bridge voltage U_g_ = ΔU. Since the measuring bridge was not adjusted to a bridge voltage U_g_ = 0 V during the measurements, ΔU is subsequently the difference between the bridge voltages with and without target gas. Catalytic gas sensors are commonly deployed within a Wheatstone bridge configuration to counterbalance disturbances that include variations in airflow, ambient temperature, and alterations in the thermal conductivity of the surrounding medium, which may occur as a result of heightened humidity levels, for instance. The so-called active pellistor is coated with a catalyst. The uncoated inert pellistor serves as a reference sensor. By applying flammable gas, the temperature of the active pellistor increases by the exothermic reaction. If the temperature rise is too high, the pellistor itself can act as an ignition source or can be damaged. In the succeeding analysis, we endeavor to compute the magnitude of absolute temperature augmentation, specifically attributed to exposure to combustible gases, given the selected material system and corresponding substrates. This computational estimation will shed light on the thermal dynamics associated with the material system under the influence of flammable gases.

The absolute temperature increase of the pellistor is calculated using the Wheatstone equation at unbalanced state of Wheatstone Bridge (1) and the Equation (2) derived from it:

(1) $$U_{g}=U_{s}\left(\frac{R_{meas}}{R_{ref}+R_{meas}}-\frac{R_{1}}{R_{1}+R_{2}}\right),$$

U_g_: bridge voltage at gas admission; U_s_: supply voltage; R_1_ = R_2_: fixed resistances in the bridge, R_ref_: resistance of the Pt heater of the uncoated ceramic substrate (reference); R_meas_: resistance of the Pt heater of the active pellistor when exposed to a combustible gas.

By setting R_1_ equal to R_2_, the following expression is obtained for the heater resistance of the pellistor:

(2) $$R_{meas}=R_{ref}\left(\frac{{2U}_{g}+U_{s}}{U_{s}-{2U}_{g}}\right),$$

Since we know the resistance value of R_ref_, we can calculate the resistance change of R_meas_ when gas is applied. Using the temperature coefficient of Pt (for the substrates used, α = 0.0382 K^−1^ which is determined by calibration experiment), the temperatures of R_meas_ and R_ref_ can be determined. Then the absolute temperature increase is the difference between T_meas_ and T_ref_.

The measurements were carried out at a gas measuring set-up with calibrated mass flow controllers and certified test gas bottles, which allows different test gas concentrations to be set. All gas measurements were performed with dry synthetic air as carrier gas for the test gases.

## 3. Gas Sensitive Characterization

Monitoring the lower explosive limit of methane-containing gas mixture is one of the main applications of catalytic sensors. Among the alkane compounds, methane (CH_4_) is the main component of natural gas and biogas. Methane serves as a foundational compound in the chemical industry, functioning as the precursor for an extensive array of technologically significant synthesis processes. Methane forms explosive mixtures at a volume fraction of between 4.4 and 16.5 percent in air [1]. Methane is highly flammable, and its ignition temperature is 595 °C [44].

In addition to methane, the sensors were also characterized using propane. The monitoring of propane is highly relevant for safety reasons as well since it serves as a liquid gas for combustion processes and as a refrigerant [45]. For example, propane is used as LPG (liquefied petroleum gas) for driving vehicles, in gas stoves, gas boilers, gas grills, and welding equipment. Generally, propane is mixed with butane. As a refrigerant [46], propane has the designation R-290 and it is used in refrigerators and heat pumps [47]. In Australia, propane is now used in the majority of vehicle air conditioning systems [48]. In addition, propane, besides methane and ethane, has the third largest share in natural gas [49]. In contrast to methane, propane is heavier than air. Propane is highly flammable and forms an explosive mixture between 2.1% and 9.3% by volume in air [50]. The ignition temperature is 470 °C and is thus in the range of the operating temperature of commercially available pellistors. They are therefore a potential ignition source and must therefore be protected with a flame arrestor.

Gas characterization was performed using a specialized gas measurement system. This gas measurement system includes multiple subsystems, such as a gas delivery system, power supply, and read-out electronics. 

The gas atmosphere dependent behavior of the pellistors in response to test gases was investigated using a digital voltmeter (Keithley 2700), a scanner card (National Instruments) and a laboratory computer. A self-written LABVIEW program was used for data acquisition. Gas atmospheres were controlled with calibrated mass flow controllers (Bronkhorst FG-201AV). 

All gas measurements were carried out in a closed gas chamber filled consecutively with synthetic air and with the target gas of the specified concentration. The test gases were purchased from Westfalen and have a purity of 99.99. To obtain the defined target gas concentration, the gases were mixed with dry synthetic air (21% O_2_, 79% N_2_; Westfalen, Germany) as gas carrier using the calibrated mass flow controllers.

### 3.1. Detection of Methane

**Figure 6:**
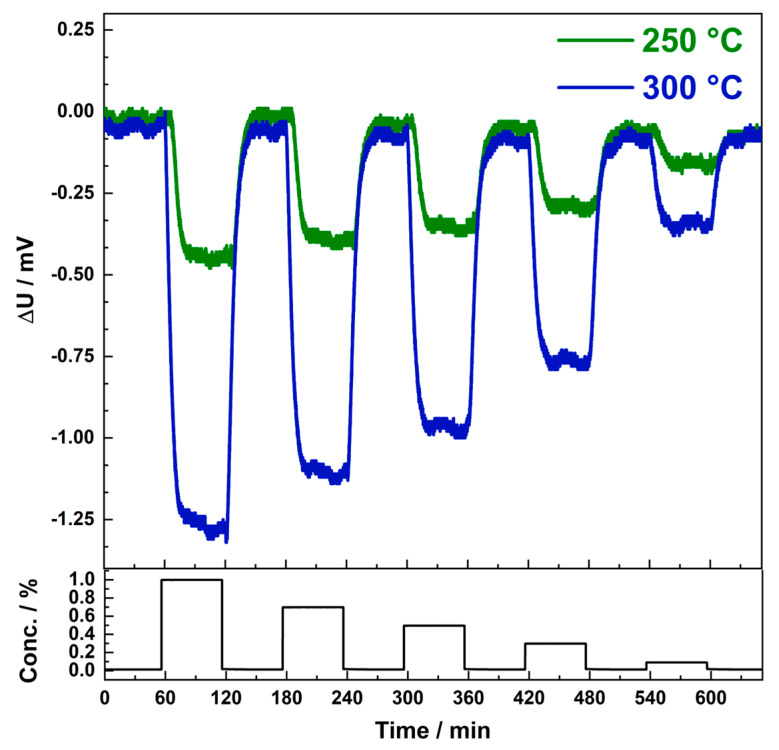
Response ΔU to methane exposure of a pellistor coated with 15 layers of Au–Pd particles (12 wt%) on mesoporous Co_3_O_4_. The pellistor was exposed to 1%, 0.7%, 0.5%, 0.3%, and 0.1% methane. The operating temperatures were 300 °C and 250 °C.

For the gas sensitive measurements, methane concentrations relevant for LEL monitoring were carefully selected. Figure 6 shows the measurements made with 0.1% to 1% methane in synthetic air at 300 °C and 250 °C operating temperature. The pellistor shows a clear, distinguishable signal for all used concentrations and at both temperatures. Already at a working temperature of 250 °C, a substantial sensor signal is detected. As evident from Figure 6, the pellistor demonstrates excellent working performance at 250 and 300 °C, which can be considered "low temperature" in comparison to conventional pellistors.

**Figure 7:**
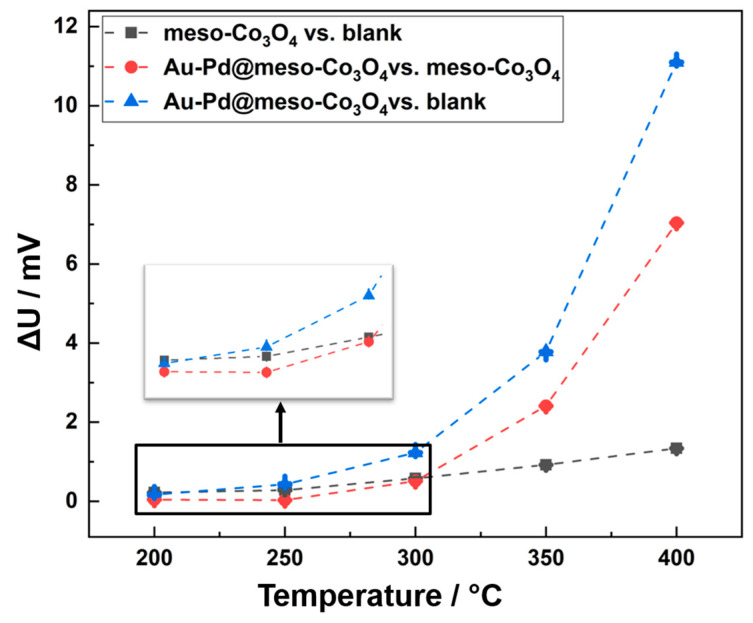
Response ΔU of different pellistors vs. different reference sensors during exposure to 1% methane and operation temperatures between 200 °C and 400 °C. The measurement Au–Pd(12 wt%)@meso-Co_3_O_4_ vs. blank sensor (blue) shows the catalytic effect of Au–Pd on mesoporous Co_3_O_4_, the measurement meso-Co_3_O_4_ vs. blank (black) the effect of blank mesoporous Co_3_O_4_ and measurement Au–Pd@meso-Co_3_O_4_ vs. meso-Co_3_O_4_ (red) the influence of the pure Au–Pd particles on the methane oxidation. The measurements show that below 300 °C the Au–Pd particles no longer have a catalytic effect. The catalytic conversion of methane comes solely from the mesoporous Co_3_O_4_.

In order to understand the individual role of Au–Pd, the pellistor made of meso-Co_3_O_4_ was tested against the blank Al_2_O_3_ substrate as reference sensor. In addition, the meso-Co_3_O_4_ pellistor was used as reference sensor instead of the blank Al_2_O_3_ substrate to test the Au–Pd(12 wt%)@meso-Co_3_O_4_ pellistor, aiming to elucidate the function of Au–Pd in the catalyst system. The results in Figure 7 show that the red curve for Au–Pd@meso-Co_3_O_4_ vs. meso-Co_3_O_4_ has higher values than the black curve for meso-Co_3_O_4_ vs. blank, indicating that Au–Pd noble metal particles dominate the catalytic combustion above 300 °C compared to Co_3_O_4_, whereas Co_3_O_4_ prevailed against Au–Pd below 300 °C. Taking the blue curve for Au–Pd@meso-Co_3_O_4_ vs. blank into account for comparison, the results suggest that despite a residue effect of Au–Pd between 250 °C and 300 °C, the main effect of the overall catalytic effect for Au–Pd@meso-Co_3_O_4_ below 300 °C mainly arises from Co_3_O_4_. Moreover, Au–Pd is supposed to be responsible for the overall pellistor performance above 300 °C.

### 3.2. Detection of Propane

**Figure 8:**
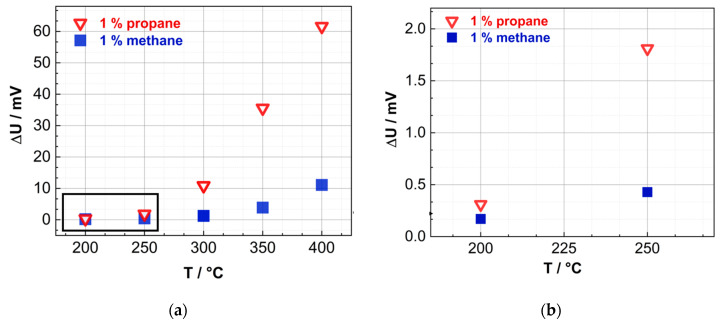
Signal of the pellistor when exposed to 1% methane and 1% propane, where the results in the black box in figure (**a**) are enlarged in (**b**). (**a**) Measured voltage U_g_ during exposure with different methane and propane concentrations plotted vs. the reference sensor operation temperature. The active sensor is coated with Au–Pd particles (12 wt%) on mesoporous Co_3_O_4_ as a carrier. The reference sensor is uncoated. (**b**) Results for operating temperatures of the reference sensor below 300 °C are shown enlarged.

The response ΔU when exposed to different methane and propane concentrations plotted against the reference sensor temperature is shown in Figure 8. The sensor response, when exposed to 1% propane and operating at 400 °C, is almost 6× greater than the sensor response to the same concentration of methane. Even at a low operating temperature of 250 °C, the pellistor signal of propane is more than 3× higher than that of methane.

This can be explained by the high reactivity and high heat of combustion of propane compared to methane [51]. Methane has a heat of combustion of 889 kJ/mol, and propane has a value of 2220 kJ/mol [51]. Therefore, propane releases approximately 2.5× more heat of combustion than methane. In addition, the consideration of heat of combustion, the reactivity effect of propane contributes to the larger sensor signal as well. The higher reactivity of propane is due to the different binding energies. The C–H bond energy in methane (CH_4_) is 439 kJ/mol, making it the strongest C–H bond in the alkane family. While in propane the primary C_3_H_7_-H binding energy is only 423 kJ/mol, the secondary (CH_3_)_2_CH-H is even weaker, 412 kJ/mol [52]. The weaker C–H bond in propane allows for a catalytic combustion taking place at lower temperature than methane.

## 4. Determination of the Absolute Temperature Increase of the Catalytically Active Layer When Exposed to Combustible Gases

As previously mentioned, catalytic gas sensors are commonly implemented in a Wheatstone bridge configuration in order to compensate for external disturbances such as changes in airflow, ambient temperature, and alterations in the thermal conductivity of the surrounding medium, which may result from heightened humidity levels. The active pellistor sensor, coated with a catalyst, is accompanied by an uncoated, inert pellistor that serves as a reference sensor. Upon exposure to combustible gases, the exothermic reaction increases the temperature of the active pellistor. However, if the temperature rise is excessive, the pellistor may either act as a source of ignition or sustain damage. In the following analysis, the absolute temperature increase is estimated due to the exposure to combustible gases for a specific material system and substrate selection.

**Figure 9:**
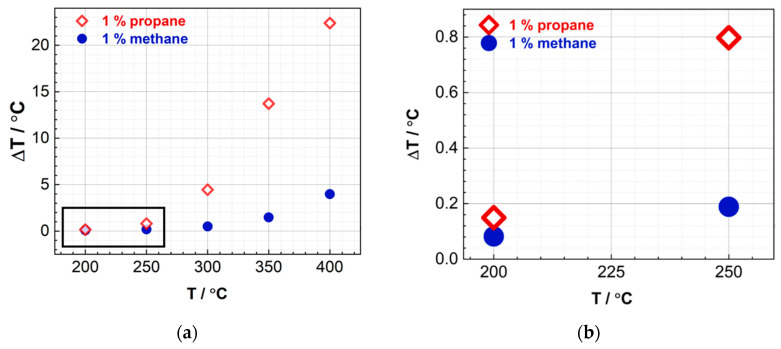
Temperature change of the pellistor when exposed to 1% methane and 1% propane, where the results in the black box in figure (**a**) are enlarged in (**b**). (**a**) Calculated temperature difference between the sensor with Au–Pd (12 wt%)@meso-Co_3_O_4_ and the reference sensor operated in a Wheatstone bridge plotted against the reference sensor temperature when exposed to 1% methane and propane in synthetic air. (**b**) Results for operating temperatures of the reference sensor below 300 °C are shown enlarged.

The temperature difference (∆T) between the pellistor and its reference sensor during the pellistor operation is of interest and has been evaluated in Figure 9. The analysis indicates a positive correlation between ∆T. It is worth noting that the temperature increase ΔT exhibits a considerable difference for propane, which has a higher susceptibility to oxidation, in comparison to methane. At a temperature of 400 °C, the temperature increase for methane is approximately 4 °C, while that of propane is approximately 22 °C. This observation is logical and in line with expectations.

Upon contact with a pellistor, propane undergoes oxidation, resulting in the release of heat and causing a corresponding increase in the temperature of the wires. Due to its higher heat of combustion, propane produces more heat when oxidized, causing the temperature of the pellistor wires to rise more than when methane is oxidized. Therefore, the temperature increase, ΔT, for propane, which is more readily oxidizable, is significantly higher when compared to methane. Based on these findings, future research could focus on developing more sensitive substrates with a larger temperature slope (ΔR/ΔT).

## 5. Conclusions

Mesoporous Co_3_O_4_ supported Au–Pd nanoparticles has been investigated with respect to its suitability for a gas sensing material to build pellistors for a reliable detection of the lower explosion limit of methane and propane. Our results suggest that the operation temperature for the pellistor using Au–Pd@meso-Co_3_O_4_ can be lowered down to at least 300 °C for methane detection. Monitoring the lower explosive limit for the more easily oxidized propane is possible even at 250 °C. The investigated material has a three-dimensional mesoporous structure, ensuring a higher surface and a high degree loading of Au–Pd, which might account for the improved sensor behavior at low temperature compared to the conventional pellistors. A high coverage of Au–Pd alloy particles on the surface of the carrier material has a positive effect on the detection of combustible gases. The measurements under propane exposure indicated a greater temperature increase, and thus a higher sensor response compared to that under methane. This can be explained by its larger heat of combustion and high reactivity arising from the lower C–H binding energy. We investigated the individual role of the Au–Pd particles and its support Co_3_O_4_.The results show that below 300 °C the Au–Pd particles no longer have a catalytic effect. The catalytic conversion of methane below 300 °C comes solely from the mesoporous Co_3_O_4_. The fact that no metallic catalyst at all is needed at an operating temperature of the pellistors below 300 °C is one of the most important findings from the investigations carried out. Our finding suggested that the future work should be focused on looking into the spinel oxide candidates. By shifting away from metallic catalysts, the envisioned outcome is an enhanced resistance to permanent catalytic poisoning, thereby ensuring greater robustness and longevity of pellistor-based systems.

## Data Availability

Not applicable.
